# 

**Published:** 1897-03-27

**Authors:** 


### EARLY EDITION.

**Notices of Births, Deaths, and Marriages** are inserted in this column at a charge of 2s. 6d. for an announcement not exceeding four lines.

### NOTICE TO CORRESPONDENTS.

All MS., Letters, Books for Review, and other matters intended for the Editor should be addressed The Editor, "The Hospital," The Hospital Building, 28 & 29, Southampton Street, Strand, W.C.
The Editor cannot undertake to return rejected MS., even when accompanied by stamped directed envelope.

**Notice to Advertisers and Subscribers.**

All Advertisements, Orders for Copies of The Hospital, and Business Communications should be addressed to **THE MANAGER, (Not to The Editor),** The Hospital, The Hospital Building, 28 & 29, Southampton Street, Strand, London, W.C.
The Cost of Subscription, post free, is as follows:—Per week, 2½d.; per quarter, 2s. 8d.; per half-year, 5s. 3d.; per annum, 10s. 6d. Foreign:—Per annum, 15s. 2d.; payable, in all cases, in advance.

**NOTICE.—Vol. XX. of "The Hospital" (April to September, 1896), in handsome cloth binding, is now on sale at the Office of this Journal, price 7s. 6d. Cases for binding the Numbers contained in this Volume, 1s. 6d. Index to Vol. XX., 2½d., post free.**

**The Monthly Part for April will be ready on April 1st. Price 6d., by post 8d.**

### Books Received.

A. Stenhouse, Glasgow.
"Forensic Medicine and Toxicology." By C. O. Hawthorne, M.B.

W. H. and L. Collingridge.
"The Wasted Orchards of England." By the *Gardeners' Magazine* Special Commissioner.

Abbott, Jones, and Co.
"A Volunteer Nurse." By Anne Thacker.

A. La Riviere.

"Sight Testing for the G.P." By F. Davidson.

Isbister and Co.
"Westminster Abbey." By the Very Rev. Dean Farrar.
"Winchester Cathedral." By the Rev. Canon Benham.
"St. Albans Abbey." By the Rev. Canon Liddell.
"York Minster." By the Very Rev. Dean Purey Cust.

Simpkin, Marshall, Hamilton, Kent, and Co.
"Mrs. Oliphant's Lie." By Keith Sandys.

James Turner and Co., Edinburgh.
"Plans of Modern Asylums for the Insane Poor." By John Sibbald, M.D.

The Rebman Publishing Co.
"Injuries and Diseases of the Ear." By Macleod Yearsley, F.R.C.S.

Sampson Low, Marston, and Co.
"Twentieth Century Practice of Modern Medical Science." Edited by Thomas Stedman, M.D. Vol. VIII.

Scientific Press.
"District Nursing." By Amy Hughes.

J. B. Lippincott and Co.
"Diseases of the Eye." By William F. Norris, M.D., and Charles A. Oliver, M.D. Vol. I.

**Periodicals and Pamphlets.**—London, St. Bartholomew's Hospital Journal, Electrical Review, Vegetarian, Industries and Iron, Literary Digest.

### Scraps and Gleanings.

The worshipful Company of Clothworkers have sent a donation of £100 to the Royal Hospital for Diseases of the Chest, City Road, E.C.

The Clothworkers' Company have given a further donation of £50 to the Italian Hospital, Queen's Square, thus raising their grant to £100.

Of the total collection on Hospital Sunday last year in Hull, the Church of England contributed £331, Wesleyan Methodists £169, Primitive Methodists £57, Congregationalists £43, Roman Catholics £38, Old Hebrew Congregation £37, Sunday schools £29, Presbyterians £15, Unitarians £12, German and Danish Lutherans £11, Society of Friends £10, various £11; total, £765.

Dr. Norman Kerr, Chairman of the Inebriates' Legislation Committee of the British Medical Association, has received a letter from Lord Salisbury confirming the intention of the Government to bring in, should time admit, a Bill for Amended Legislation for Habitual Drunkards, during the current session.

The medical superintendent of the Dundee Royal Infirmary has had to complain again of the firing of the time gun in Barrack Park, owing to the bad effect the vibration caused by it had on the patients. The Docks Committee of the Dundee Town Council, after consulting the honorary medical staff, have, however, decided not to discontinue it.

### The Student of Medicine.

### APPOINTMENTS.

C. J. Arkle, M.D.Lond., L.R.C.P., appointed Assistant Physician to the Hospital for Consumption and Diseases of the Chest, Brompton. A. S. Barling, M.R.C.S., L.R.C.P., appointed Honorary Surgeon to the Royal Lancaster Infirmary. G. A. Barrow, M.R.C.S., L.R.C.P.Lond., appointed Medical Officer to the Out-Patient Department of the Children's Hospital, Manchester. J. Cullen, L.R.C.P., L.R.C.S.I., appointed Medical Officer for the Castleknock Dispensary District. R. N. Fernandes, M.B., C.M.Edin., appointed Medical Officer for the Acworth District of the Pontefract Union. E. A. Gibson, M.B., C.M.Glasg., L.M.Rotunda, appointed Obstetric Physician to the Glasgow Training Home for Nurses. C. Jeffrey, M.B., C.M. Aberd., appointed Medical Officer for the Altofts District of the Wakefield Union. J. O. Jones, M.B., C.M.Edin., appointed Medical Officer of the Workhouse of the Holywell Union. S. L. Jones, M R.C.S , L.R.C.P., appointed Junior Medical Officer of the London County Asylum, Colney Hatch, N. A. E. Joscelyn, M.R.C.S., L.R.C.P. L.S.A., appointed Medical Officer with charge of out-patients to the Miller Hospital, Greenwich. W. Lake, M.R.C.S.Eng., L.S.A., D.P.H.Camb., Medical Officer of Health of the Guildford and Woking Districts of West Surrey, appointed Deputy Coroner for West Surrey. G. A. Leon, L.R.C.P., M.R.C.S., appointed Honorary Medical Officer to the Sidmouth Cottage Hospital, also Medical Officer to the Sidmouth Dispensary and Rational Sick and Burial Club. H. Littlejohn, M.A., M.B., B.Sc., F.R.C.S.Edin., appointed Lecturer on Medical Jurisprudence and Public Health at the School of Medicine, Surgeon's Hall, Edinburgh. W. Millar, M.B., C.M.Glas., appointed Medical Officer for the West District of the Edmonton Union. W. B. Morton, M.D.Lond., L.R.C.P., M.R.C.S.Eng., appointed Resident Medical Officer at Brislington House, Bristol. J. A. Nealon, M.D.R.U.I., appointed Medical Officer of the Workhouse and the Linton District of the Linton Union. G. Newman, M.D.Edin., D.P.H.Camb., appointed Medical Officer of Health for the Parishes of Charterhouse, Furnival's Inn, and Staple Inn. H. V. Palin, M.B., C.M., L.R.C.P., L.R.C S.Edin., L.S.A.Lond., appointed Public Vaccinator of No. 3 District Wrexham Union. T. Partridge, M.R.C.P.I., M.R.C.S., appointed Medical Officer of Health to the Nailsworth Urban District. G. S. Pope, L.R.C.P.& S.Edin., Assistant Medical Officer, Cane Hill Asylum, appointed Medical Superintendent of the New Middlesbrough Asylum, Marton, Yorkshire. H. A. Scott, M.B.& Ch.B.Vict., M.R.C.S., L.R.C.P., appointed Junior House Surgeon to the Ancoats Hospital, Manchester. Dr. Simpson, appointed Medical Officer for the Coningsby District of the Boston Union. D. Stanley, M.D.Edin., M.R.C.P.Lond., appointed Extra Acting Physician to the Children's Hospital, Birmingham. R. Stobo, M.B., C.M Glas., appointed Medical Officer of Health to the Sunderland Rural District Council. G. P. Tomey, B.A., L.R.C.S.I., L R.C.P.I., L.M., appointed Medical Superintendent, Lincolnshire County Asylum. A. Tuxford, M.B.Edin., appointed Medical Officer of Health to the Boston Rural District Council. J. J. Wallace, M.B., B.ChR.U.I., appointed Medical Officer for the Workhouse and the Weobley District of the Weobley Union.

### WORKS FOR NURSES.

**A MANUAL FOR HOSPITAL NURSES** AND OTHERS ENGAGED IN ATTENDING ON THE SICK. By EDWARD J. DOMVILLE, L.R.C.P., M.R.C.S. Eighth Edition. Crown 8vo., 2s. 6d.

**A MANUAL OF NURSING, MEDICAL AND SURGICAL**, By C. J. CULLINGWORTH, M.D., F.R.C.P. Third Edition. Fcap. 8vo., 2s, 6d.

**A SHORT MANUAL FOR MONTHLY NURSES.** By C. J. CULLINGWORTH, M.D. Fourth Edition. Revised by M. A. Atkinson. Fcap. 8vo., 1s. 6d.

**NOTES ON GYNÆCOLOGICAL NURSING.** By J. B. HELLIER, M.D. Fcap. 8vo., 1s. 6d.

**HINTS ON ELEMENTARY PHYSIOLOGY.** By FLORENCE A. HAIG-BROWN. With a Preface by Dr. Ord. With 21 Illustrations. Fcap. 8vo., 1s. 6d.

**LECTURES ON MEDICINE TO NURSES.** By HERBERT E. CUFF, M.D. With 25 Illustrations. Crown 8vo., 3s. 6d.

**ANTISEPTIC PRINCIPLES FOR NURSES.** By C. E. RICHMOND, F.R.C.S. Fcap. 8vo., 1s.

**A SHORT DICTIONARY OF MEDICAL TERMS.** 2s. 6d.

**CHAVASSE'S ADVICE TO A MOTHER** ON THE MANAGEMENT OF HER CHILDREN, and on the Treatment on the Moment of their more pressing Illnesses and Accidents. Fourteenth Edition (230th Thousand). 2s. 6d.

**CHAVASSE'S ADVICE TO A WIFE ON** THE MANAGEMENT OF HER OWN HEALTH, and on the Treatment of Some of the Complaints incidental to Pregnancy, Labour, and Suckling. With an Introductory Chapter especially addressed to a Young Wife. Thirteenth Edition (280th Thousand). 2s. 6d.

London: J. and A. CHURCHILL,
7, Great Marlborough Street.

